# The Frequency of Human Papillomavirus Positivity in Iranian Patients with Head and Neck Squamous Cell Carcinoma

**DOI:** 10.30699/ijp.2020.119344.2300

**Published:** 2020-10-10

**Authors:** Mitra Rezaei, Mahboobeh Karimi Galougahi, Azin Kheradmand, Mihan Pourabdollah Toutkaboni, Hassan Mir Mohammad Sadeghi, Alireza Abdollahi, Amirnader Emami Razavi, Ali Safavi Naini, Farahnaz Bidari- Zerehpoosh

**Affiliations:** 1 *Virology Research Center, National Research Institute of Tuberculosis and Lung Diseases, Shahid Beheshti University of Medical Sciences, Tehran, Iran*; 2 *Department of Pathology, School of Medicine, Shahid Beheshti University of Medical Sciences, Tehran, Iran*; 3 *Tracheal Research Center, National Research Institute of Tuberculosis and Lung Diseases, Shahid Beheshti University of Medical Sciences, Tehran, Iran*; 4 *Respiratory Research Center, National Research Institute of Tuberculosis and Lung Diseases, Shahid Beheshti University of Medical Sciences, Tehran, Iran*; 5 *Oral and Maxillofacial Surgery Department, Shahid Beheshti University of Medical Sciences, Tehran, Iran*; 6 *Imam Hospital Complex, Tehran University of Medical Sciences, Tehran, Iran*; 7 *Iran National Tumor Bank, Cancer Institute, Tehran University of Medical Sciences, Tehran, Iran*; 8 *Department of ENT, National Research Institute of Tuberculosis and Lung Disease, Tehran, Iran*; 9 *Department of Pathology, Loghman Hakim Hospital, Shahid Beheshti University of Medical Sciences, Tehran, Iran*

**Keywords:** Human papillomavirus, Squamous cell carcinoma, Iran

## Abstract

**Background & Objective:**

Human papillomavirus (HPV) has been associated with prognosis in patients with head and neck squamous cell carcinoma (HNSCC). Similar to the global studies, different prevalence rates of this viral infection have been reported in Iran. Therefore, we aimed to report the prevalence of this virus and its significance in HNSCC patients.

**Methods:**

Patients who were referred to the five hospitals of Tehran city from May 2018 to May 2019 were enrolled in this study. All patients were diagnosed with HNSCC based on pathologic study. The pathologic disease staging was defined, and DNAs were extracted from the fresh tissue samples via kits. After polymerase chain reaction (PCR), HPV positive samples were evaluated for determining genotypes and data analysis.

**Results:**

Of the 46 patients, three patients (6.5%) showed positive HPV results with the following subtypes: 18 (in two patients), 52 (in three patients), 61 (in two patients), 67, and 73.

Comparison of variables between the groups with and without HPV showed a significant difference based on the tumor’s lymphatic invasion (*P*=0.041), peripheral lymph node involvement (*P*=0.008), and histologic grade (*P*=0.011), but no statistically significant difference in terms of other variables such as age, primary tumor site, size, pathologic stage, vascular or perineural invasion, metastasis, smoking, and alcohol consumption was found.

## Conclusion:

HPV positivity may be an important contributing factor in the lymphatic invasion, peripheral lymph node involvement, and histologic grade of cases with HNSCC and should be further investigated for its effect on prognosis.

## Introduction

Malignancies are currently the main cause of death worldwide. Among them, squamous cell carcinoma (SCC) is one of the ten top causes of cancer incidence and death (1). Head and neck squamous cell carcinoma (HNSCC) includes upper respiratory and digestive tract. The diagnosis and treatment may differ based on the tumor site, among which HNSCC of oral cavity, oropharynx, hypopharynx, and larynx are of greater importance (2). Worldwide reports indicate HNSCC in about 5% of all cancers, while in Iran it appears to be responsible for 1% of all cancers (3,4,5). 

The different incidence of HNSCC in different countries is attributed to the difference in the frequency of risk factors of this cancer, as HNSCC is recognized as a multifactorial disease. Although tobacco and alcohol consumption have been identified to increase the risk of HNSCC significantly (6), in 25% of HNSCC cases, such a relation cannot be found (7). Human papillomavirus (HPV) is a DNA virus that infects the skin and mucosal membranes with more than 100 subtypes (8). HPV infection has been suggested to increase the risk of HNSCC, independent of traditional risk factors such as tobacco abuse, alcohol consumption, genetic factors, and immunosuppression (3,9). On the other hand, HPV-related HNSCCs are usually high–grade, lymph node-positive tumors that mainly originate from the oropharynx (10). 

A recent study on the significance of HPV positivity on HNSCC severity has considered it as a more important factor than traditional prognostic markers such as tumor grade and histological subtype. Regarding its significance on prognosis, the patients may be categorized into high- and low-risk groups based on HPV positivity (11). Accordingly, it is currently advised that the positivity of HPV should be assessed in every patient with HNSCC to differentiate between HPV-related and -unrelated diseases (12).

In recent years, several studies among the Iranian population have investigated the role of HPV as a predisposing factor in SCC. Among them, the presence of HPV in esophageal SCC has been extensively studied (13). However, studies investigating this relationship in the head and neck area have been limited to the tongue (14) and oral cavity (15). This paper aimed to study the presence of HPV in SCCs in a broader spectrum of locations in the head and neck area.

##  Materials and Methods

Study Design

All patients who referred to Masih Daneshvari, Bahman, Ayatollah Taleghani, Loghman Hakim, and Imam Khomeini Hospitals (located in different parts of Tehran, Iran) during one year (May 2018–May 2019) were enrolled in this study if they were diagnosed with HNSCC by the pathologist, according to the pathologic results. Patients with a history of radiotherapy, chemotherapy, and immunotherapy were excluded from this paper.

This study was approved by the Ethics Committee of Shahid Beheshti University of Medical Sciences (code: IR.SBMU.MSP. REC.1397.328). Before the recruitment of patients into the study, the researchers explained the design and objectives of the study to all participants and asked them to read and write the written informed consent for participation in the study. They were clarified that they were free to leave the study whenever they wished to; no additional costs were imposed on the participants, and their lack of participation would not affect their treatment process. Due to financial limitations, we evaluated all patients referred to these 5 hospitals during only one year (May 2018–May 2019).

The fresh specimens added into RNAlater (Gen All Co, Shiheung, South Korea) and sent to the Central Laboratory of Shahid Beheshti Medical University for PCR. The samples were kept at 4^o^C refrigerator until theywere sent to the laboratory. DNA was extracted via the QIAamp DNA Mini Kit (QIAGEN, Hilden, Germany) according to the manufacturer’s instructions. To evaluate the quality of the extracted samples, Nanodrop (Biotek Co, Winooski, VT, USA) was used. Then, universal primers were used for PCR. PCR products were used for genotyping, performed using INNO-LiPA assay (Fujirebio Europe N.V., Gent, Belgium), based on reverse hybridization, during which, one part of L1 region (SPF10 from HPV genome) is developed by the primer. 

Then, biotinylated amplicones were hybridated with oligonucleotide probes. One pair of primers was added from the human HLA-DPB1 gene for confirming the quality of the extracted DNA. Next, streptavidin-conjugated alkaline phosphatase was added. After incubation with BCIP/NBT chromogen, the result was observed visually. Then, the samples were sequenced and used for analysis.

Statistical Analysis

Results were presented as mean ± standard deviation (SD) for quantitative variables and summarized by frequency (percentage) for categorical variables. The Kolmogorov–Smirnov test was used to assess the normal distribution of data. Continuous variables were compared by using the *t-*test or Mann–Whitney U test, whenever the data did not appear to have normal distribution or when the assumption of equal variances was violated across the study groups. On the other hand, categorical variables were compared by using the chi-square test. For the statistical analysis, the statistical software SPSS 21.0 (IBM Corp., Armonk, NY, USA) was used. P-values of 0.05 or less were considered statistically significant.

## Results

A total of 46 patients were included in this research, of whom 20 patients (43.5%) were female, and 26 patients (56.5%) were male. The mean age of participants was 61.67 ± 14.41 years. The patients’ ethnicity was Persian in 25 patients (54.3%), Azari in 12 patients (26.1%), Gilaki and Mazani in three patients (6.5%), Lur in one patient (2.2%), Baluch in one patient (2.2%), and Arab in two patients (4.3%). Seventeen patients (37%) were from Tehran, and others were from other cities. Only one patient reported alcohol drinking, and four were current smokers (four were ex-smokers).

The frequency of the primary HNSCC site was the tongue in 14 patients (30.4%), lip in 10 patients (21.7%), larynx in five patients (10.9%), mouth floor in four patients (8.7%), buccal mucosa in three patients (6.5%), hard palate in three patients (6.5%), lower gum in two patients (4.3%), pharynx in two patients (4.3%), glottis, maxillary sinus, and nasal cavity each in one patient (2.2%).

The histology grade was I (well-differentiated) in 19 patients (41.3%), II (moderately differentiated) in 22 patients (47.8%), III (poorly differentiated) in three patients (6.5%), and unknown in two patients (4.3%). Six patients (13.04%) had lymphatic invasion, five patients (10.9%) had vascular invasion, and 18 patients (39.1%) had perineural invasion. There was no case of synchronized tumors.

Three patients (6.5%) were diagnosed as HPV positive. HPV subtypes by using reverse hybrid genotyping and risk categorization are shown in Table 1.

**Table 1:** HPV subtypes and risk categorization

Patients	HPV subtypes
**Patient 1**	61 (low risk) 18 (high risk) 52 (high risk)
**Patient 2**	67(unknown) 61 (low risk) 18 (high risk) 52 (high risk) 73 (probable high risk)
**Patient 3**	52 (high risk)

Comparison of variables between the groups with and without HPV showed that all three patients with positive HPV were male, but there was no statistically significant difference in this regard (*P*=0.116) (Table 2). Also, there was no difference in the frequency of positive HPV in terms of patients’ ethnicity (*P*=0.103), place of residence (*P*=0.243), smoking status (*P*=0.170), alcohol consumption (*P*=0.065), weight loss (*P*=0.905), and primary tumor site (*P*=0.297). 

**Table 2:** The frequency of different variables based on positivity of HPV in the studied patients

		Total	Positive HPV	Negative HPV	P–value^*^
**Sex**	Female	20(43.5%)	0	20(46.5%)	0.116
	Male	26(56.5%)	3(100%)	23(53.5%)	
**Histologic grade**	Grade I	19(41.3%)	0	19(44.2%)	0.011
	Grade II	22(47.8%)	1(33.33%)	21(48.8%)	
	Grade III	3(6.52%)	1(33.33%)	2(4.65%)	
	Grade X	2(4.34%)	1(33.33%)	1(2.32%)	
**Lymphatic invasion**	Yes	6(13.04%)	2(66.66%)	4(9.3%)	0.041
	No	40(86.95%)	1(33.33%)	39(90.7%)	
**Vascular invasion**	Yes	5(10.9%)	1(33.3%)	4(9.3%)	0.196
	No	41(89.1%)	2(66.66%)	39(90.7%)	
**Perineural invasion**	Yes	18(39.13%)	1(33.33%)	17(39.53%)	0.831
	No	28(60.86%)	2(66.66%)	26(60.46%)	
**Alcohol consumer**	Yes	1(2.2%)	1(33.33%)	0	0.065
	No	45(97.82%)	2(66.66%)	43(100%)	

^*^The results of Chi-square test were considered significant at <0.05

Further, there was no difference in the frequency of HNSCC regarding pathologic stage (*P*=0.0386), vascular invasion (*P*=0.196), perineural invasion (*P*=0.831), and metastasis (*P*=0.205); meanwhile, there was a significant difference regarding the tumor’s lymphatic invasion (*P*=0.041), peripheral lymph node involvement (*P*=0.008), and histologic grade (*P*=0.011) (Table 2). The frequency of pathologic stage and peripheral lymph node involvement and their difference based on HPV positivity are shown in Table 3.

**Table 3:** The frequency pathologic T and N grades based on positivity of HPV in the studied patients

		Total	Positive HPV	Negative HPV	P–value^*^
Pathologic stage (pathologic T)	**Tx**	1(2.2%)	0	1(2.32%)	0.822
	**T1**	6(13.04%)	1(3.33%)	5(11.62%)	
	**T2, NOS**	8(17.4%)	0	8(18.6%)	
	**T2a**	1(2.2%)	0	1(2.32%)	
	**T3**	16(34.8%)	2(66.66%)	14(32.55%)	
	**T4, NOS**	1(2.2%)	0	1(2.32%)	
	**T4a**	11(23.9%)	0	11(25.6%)	
	**T4b**	2(4.34%)	0	2(4.65%)	
Peripheral lymph node involvement	**Nx**	1(2.2%)	0	1(2.32%)	0.008
	**N0**	33(71.73%)	1(33.3%)	32(74.4%)	
	**N1, NOS**	1(2.2%)	0	1(2.32%)	
	**N1a**	1(2.2%)	1(33.3%)	0	
	**N2, NOS**	1(2.2%)	0	1(2.32%)	
	**N2b**	9(19.56%)	1(33.3%)	8(18.6%)	

^*^The results of Chi-square test were considered significant at <0.05

The mean age of patients with HPV was 53.33 ± 11.71 years, and that of patients without HPV was 62.25 ± 14.52 years, while there was no statistically significant difference between the groups based on the results of the Mann–Whitney U test (*P*=0.256). Mean tumor size in patients with HPV was 4.60 ± 2.62 cm, and that of patients without HPV was 4.20 ± 2.23 cm, while there was no statistically significant difference between the groups based on the results of the Mann–Whitney U test (*P*=0.738).

## Discussion

In this study, we examined HPV positivity in patients with HNSCC from different tumor sites, sizes, and grades. The most seen tumor site was the tongue, followed by lip and buccal mucosa, as well as larynx and mouth floor. The top three frequent locations detected in our study population are locations that expected according to available data for the Iranian population (3). 

Studying the HPV positivity in our study showed that HPV was present in only three patients (all male), which equals to 6.5% of the study population. Previous studies on the prevalence of HPV between patients with head and neck, oral cavity, and oropharyngeal SCC in Iran have reported highly variable values, i.e., 21.6–60% in Southwest (16, 17), 14% in South (14), and 20–57.1% in Northeast (18,19). Even in Tehran, the recorded prevalence rates (3.2–62.5%) have followed this variable trend (20–22). 

This great discrepancy in the HPV positivity rates among patients with HNSCC may be attributable to the different techniques of HPV detection and reporting (23), different methods of patient allocation, and different ethnicity. Though our study was implemented in Tehran, the majority of our patients were referred from other cities with different ethnicities, which can be affected by the prevalence in the community of origin.

On the other hand, it seems that age has a noticeable direct effect on the prevalence of HPV positive HNSCCs. For example, in East Azerbaijan, Halimi and Morshedi (18) reported a prevalence rate of 20% with the mean age of 68.9 years, while Kermani *et al.* (19) found a prevalence rate of 57.1% with the mean age of 39.7 years. Similarly, in Tehran, Haratian *et al.* (20) reported a prevalence rate of 62.5% with the mean age of 44.37 years, while Seraj *et al.* (21) found a prevalence rate of 26.6% with the mean age of 57.88 years, and Karbalaie Niya *et al.* (22) reported a prevalence of as low as 3.2% with the mean age of 60.5 years. 

The low frequency found in a study by Karbalaie Niya *et al.* was speculated to be a result of using paraffin-embedded blocks (22). To our knowledge, our research is the only study conducted in Iran in which fresh specimens were used to detect HPV in HNSCC that is considered as the gold standard (24). The low incidence found in our study is in accordance with Karbalaie Niya *et al.* and shows that using paraffin-embedded blocks may not be the reason.

In the Islamic Republic of Iran, the law obliged the citizens to have sex within the framework of Islamic law, which forbids extramarital sexual relationships. However, based on unofficial records and observations the percentage of the youth and younger generation who disobey these regulations and engage in extramarital sexual activities seems to be increasing. Also, the number of people getting married at a young age (and therefore stay in a single-partner relationship) is declining (25). This changing sexual behavior may increase HPV transmission, like other sexually transmitted diseases (STDs), in the younger generation. 

On the other hand, it has been shown a significant association between HPV DNA positivity and alcohol consumption among patients with HNSCC (26). Smoking may also have a synergistic effect on HPV persistence when combined with alcohol drinking (27). The growing trend of smoking and alcohol consumption among the Iranian younger generation may explain the noticeable higher prevalence in younger target populations than in the older population, such as our patients.

In our study, comparing the clinical and pathologic variables, among HPV-positive and HPV-negative groups, showed no difference in terms of patients’ sex, age, ethnicity, place of residence, smoking status, alcohol consumption, weight loss, primary tumor’s site, size, pathologic stage, vascular invasion, perineural invasion, and metastasis, while there was a significant difference based on the tumor’s lymphatic invasion, lymph node involvement, and histologic grade.

Although a better survival rate in HPV positive HNSCC was reported by a couple of previous studies (15), the majority of studies, which investigated the prognostic role of HPV positivity in HNSCC patients, found it highly correlated with poor tumor-cell differentiation, which may act as a prognostic marker and play a vital role in the pathways that enhance lymphatic metastasis (28,29). A similar result was also reported in Iran with a higher mortality rate in HPV positive HNSCC (15). In addition, there was a significant association between HPV positivity and the grade of the primary tumor (30,31). Interestingly, these studies could not find a significant correlation between HPV positivity and other prognostic markers, such as perivascular or perineural invasion, while associated with the overall survival rate (15,23,29). 

HPV16 is believed to be the most prevalent HPV genotype in HNSCC worldwide (22). It was frequently detected in Iranian studies followed by HPV18 (19,20,22). In our study, there was found no HPV16 genotype, but two patients were carriers of HPV18.

Undeniably, there are some limitations of our study which have to be addressed. The main limitation was our small sample size to scrutinize the wide spectrum of HNSCCs. Although it offered a chance to generalize the findings to all HNSCCs, as HPV positivity is dependent on the tumor location in different locations of head and neck and oropharyngeal areas, it declined the number of HPV positive tumors. Furthermore, we did not randomize patients for their enrollment into the study or groups, increasing the chance of confounders on the results.

## Conclusion

In conclusion, our study showed a positive status for HPV in 6.5% of all patients with HNSCC. This value, which is the least reported compared with other Iranian studies, suggests a significant influence of age on the prevalence of this infection among the Iranian population. Observing the significant correlation of HPV-positive HNSCC with lymph node involvement reaffirms the need for determining HPV infection status in risk stratification of patients and subsequent more aggressive treatment protocols. Further prospective studies are required to investigate the accurate effect of HPV on each tumor site of HNSCC and different ages in a larger sample size.
